# Rugoscopic Evaluation of Periodontal Disease: A Cross-Sectional Study

**DOI:** 10.7759/cureus.66545

**Published:** 2024-08-09

**Authors:** Amrutha Sairam, Viola Esther, Ravishankar PL, Kalaivani V, Rajapandian K, Sindhuja R, Mohamed Rashik, Merita Stanley, Sanjana J

**Affiliations:** 1 Department of Periodontology, SRM Kattankulathur Dental College and Hospital, SRM Institute of Science and Technology, Chengalpattu, IND

**Keywords:** calcorrugoscopy, rugae, chronic gingivitis, aggressive periodontitis, chronic periodontitis

## Abstract

Introduction

Rugoscopy, which is the study of the ridges on the roof of the mouth, or rugae palatinae, is frequently employed to identify individuals. Since these ridges maintain their shape throughout a person's life, their unique patterns are used as a diagnostic aid for periodontal diseases.

Objective

The aim of this study is to explore the connection between palatal rugae patterns and periodontal diseases.

Methodology

A study group of 120 patients aged between 18 and 60 was meticulously grouped according to the American Academy of Periodontology (AAP) 1999 classification of periodontal diseases: Group A comprised healthy individuals, Group B had patients diagnosed with gingivitis, Group C included patients diagnosed with chronic periodontitis, and Group D had patients diagnosed with aggressive periodontitis. Maxillary impressions from each group were cast in dental stone and analyzed in detail for rugae patterns.

Results

Patients with gingivitis and chronic periodontitis predominantly showed a sinuous rugae pattern, while those with aggressive periodontitis exhibited an angular rugae pattern. Using the chi-square test (p < 0.05) to perform statistical analysis confirmed a significant association between rugae patterns and diagnosis, thus supporting the hypothesis of a nuanced relationship between palatal rugae patterns and specific periodontal conditions.

Conclusion

In summary, palatal rugoscopy provides a sophisticated and precise method for identifying and understanding various periodontal conditions, offering a potential breakthrough in diagnosing and prognosticating these conditions.

## Introduction

Periodontal diseases, which are multifactorial conditions encompassing gingivitis and periodontitis, are widespread diseases that affect individuals of all age groups globally. These diseases primarily affect the tissues surrounding and supporting the teeth. Early detection and precise diagnosis of periodontal diseases are of the utmost importance to prevent their progression. If left untreated, these conditions can lead to tooth loss and contribute to systemic health issues such as cardiovascular disease and diabetes. The current methods for diagnosing periodontal diseases rely on a combination of clinical assessment, radiographic imaging, and laboratory tests [1]. Albeit being widely used, these diagnostic methods do not forecast the onset or progression of periodontal diseases.

Rugoscopy, which examines the rugae palatinae, which are the ridges on the roof of the mouth, can be used as a potential predictive device due to the genetic predisposition of these patterns, as there is no established method to predict and prevent periodontal disease. Palatal rugoscopy, or palatoscopy, is a method of studying palatal rugae or folds present on the hard palate, often used as adjuncts to forensic odontology and anthropology [2]. Palatal rugae patterns are unique to each individual, similar to their fingerprints. Allen et al. (1889) were the first to use palatal rugae for personal identification [3]. Later, EI-Sharkawy (1998) reported that palatal rugae patterns are sufficiently distinctive to differentiate individuals [4]. In 2009, Filho et al. [5] demonstrated through their research that rugae patterns are as unique as fingerprints and retain their shape throughout life, making them valuable for forensic odontology. Palatal rugae's distinctive patterns and uniqueness are well recognized in various dental fields [6,7]. The stability of palatal rugae and their association with periodontal disease can aid in early detection, potentially leading to better prognoses, reduced need for surgical interventions, and prevention of long-term negative impacts on mental well-being [8]. Given the lack of sufficient studies on this topic, our research aimed to leverage palatal rugae patterns to explore their potential association with the diagnosis of periodontal disease. The study's objectives were to identify the number and pattern of palatal rugae across different periodontal disease groups and to determine if the rugae pattern is distinctive enough to be considered a predictive factor for identifying periodontal disease.

## Materials and methods

A prospective cross-sectional study was conducted at a single center on 120 patients aged 18-60 years at the outpatient department of periodontics, SRM Kattankulathur Dental College and Hospital, Chengalpattu, India. The selected subjects, who met the inclusion criteria, were divided into four equal groups of thirty patients. Approval for the study was obtained from the institutional ethics committee (approval number: SRMIEC-STO324-979), and consent was obtained from all the participants after providing sufficient information regarding the study. The participants were selected based on specific inclusion criteria: adults aged 18 to 60 years, including both males and females, who were in good general health. Additionally, participants had to have periodontal disease as classified by the American Academy of Periodontology (AAP) classification of 1999 [9]. Exclusion criteria for the study included patients with a history of palate surgery, those undergoing orthodontic treatment, and individuals with palatal anomalies. Additionally, patients with palatal cysts, ulcers, fractures, or a gag reflex were excluded from participation.

Sample size estimation

The sample size was estimated by ANOVA software, and the required sample size was estimated as 120 with 30 in each group. The probability of observing a score is 0.35 (X>Y) with 80% power and 5% acceptance of error level (∞).

Subjects were divided into four groups according to the 1999 AAP classification [9], with each group defined by their recorded diagnoses: Group A: periodontally healthy group; Group B: patients diagnosed with gingivitis; Group C: patients diagnosed with chronic periodontitis; Group D: patients diagnosed with aggressive periodontitis.

**Figure 1:**
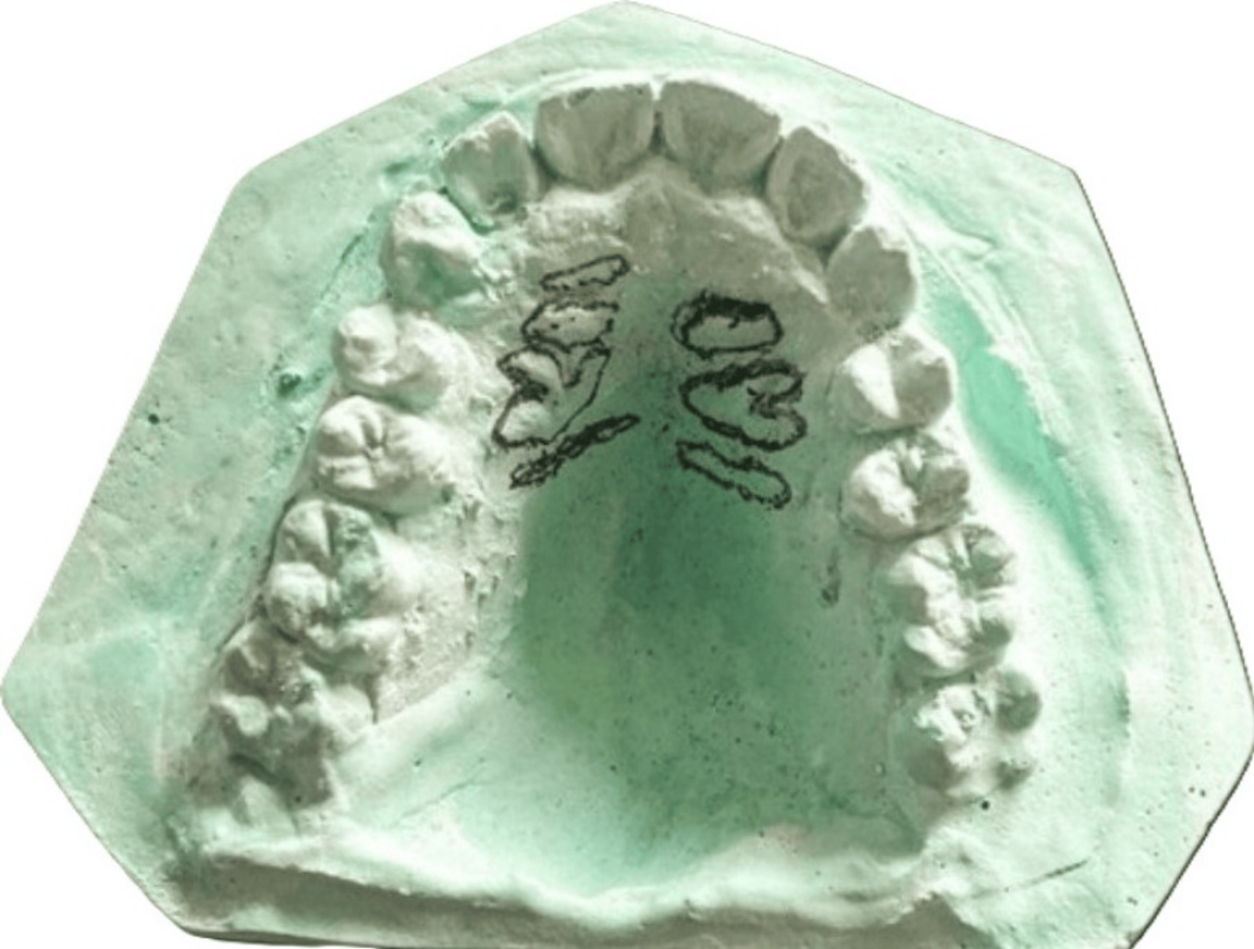
Palatal rugae markings on die stone cast

**Figure 2:**
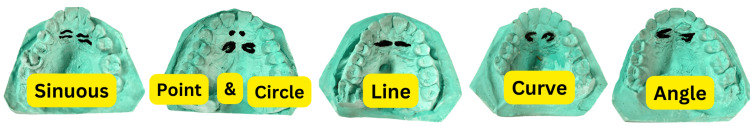
Classification of the rugae patterns according to Trobo et al. Source:  [10]

Impressions of the maxillary arches of participants were taken with alginate impression material using stock trays, and casts were created using dental stone model Type 3 following the instructions given by the manufacturer, as shown in Figure 1. The raised ridges on the cast, depicting palatal rugae patterns, were carefully marked with a lead pencil and examined according to the approach used by Trobo et al. (1932) [10], as elaborated in Figure 2.

## Results

Statistical analysis

The sample size was estimated by ANOVA software, and the required sample size was estimated as 120 with 30 in each group. The probability of observing a score is 0.35 (X>Y) with 80% power and 5% acceptance of the error level (∞).

The collected data underwent statistical analysis utilizing the chi-square test. With a p-value calculated at 0.00088, which is considerably below the set level of significance p <0.05, the chi-square test underscored a notable correlation between rugae patterns and diagnosis. This suggests that rugae patterns could potentially serve as valuable supplementary diagnostic markers for distinguishing between various forms of periodontal diseases.

The research hypothesis (H1) posits that there is a dependence between the rugae pattern and diagnosis. Conversely, the null hypothesis (H0) suggests that the rugae pattern and diagnosis are independent. To validate the research hypothesis, we must show that the rugae pattern and diagnosis are actually dependent.

Table 1 summarizes the chi-square test results used to correlate rugae patterns with periodontal diagnosis.

**Table 1:** Chi-square test to correlate the rugae and periodontal diagnosis The analysis has 12 degrees of freedom. The calculated chi-squared value is 33.27, while the tabulated value at a significance level of p = 0.05 is 21.03. The resulting p-value is 0.00088, which is compared against the significance threshold of 0.05. As the calculated chi-square value exceeds the tabulated value at p = 0.05, the null hypothesis is rejected, indicating a dependency between the rugae pattern and diagnosis.

Observed values	Healthy	Gingivitis	Chronic periodontitis	Aggressive periodontitis
Line	9	7	5	2
Sinous	5	14	10	6
Angle	2	5	11	14
Point	5	2	2	1
Curve	9	2	2	7
Column total	30	30	30	30
Expected values	Healthy	Gingivitis	Chronic periodontitis	Aggressive periodontitis
Line	5.75	5.75	7.09	4.41
Sinous	9.5	9.5	11.72	7.28
Angle	7.75	7.75	9.56	5.94
Point	2.5	2.5	3.08	1.92
Curve	4.5	4.5	5.55	3.45
Chi^2 ^	Healthy	Gingivitis	Chronic periodontitis	Aggressive periodontitis
Line	1.84	0.27	0.17	1.31
Sinous	2.13	2.13	0.14	0.22
Angle	4.27	0.98	0.165	4.64
Point	2.5	0.1	0.37	0.44
Curve	4.5	1.39	1.07	0.45

**Figure 3:**
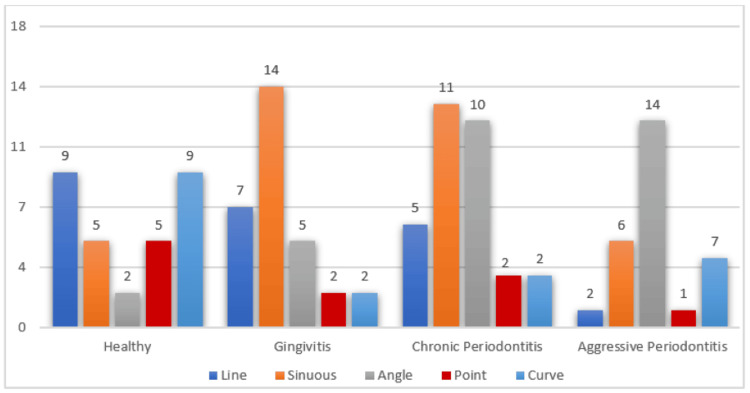
A bar graph illustrating the various types of palatine rugae patterns observed in healthy individuals and in patients diagnosed with gingivitis and periodontitis, both chronic and aggressive

Among healthy individuals, there was a significant predominance of linear and curved rugae patterns, contrasting with the more frequent presence of sinuous rugae patterns in patients diagnosed with chronic periodontitis and gingivitis. Conversely, angular patterns were predominantly observed in patients diagnosed with cases of aggressive periodontitis, as illustrated in Figure 3.

**Figure 4:**
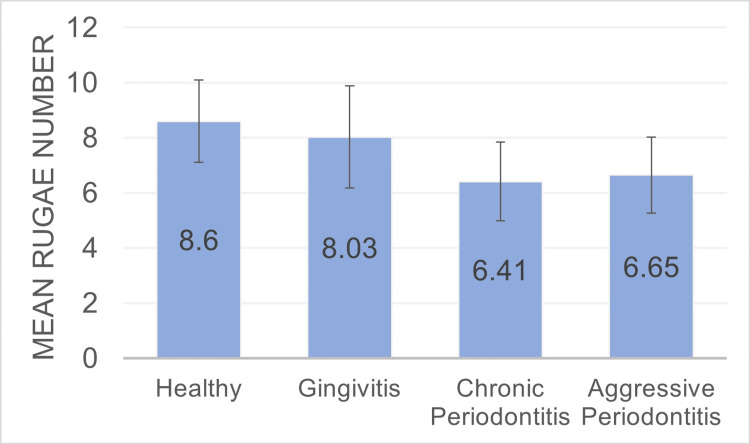
A bar graph presenting the total mean rugae number in subjects.

Our study revealed that the mean average number of rugae varied across different periodontal conditions. Specifically, the counts were as follows: 6.65 ± 1.38 for aggressive periodontitis, 6.41 ± 1.43 for chronic periodontitis, 8.03 ± 1.85 for gingivitis, and 8.6 ± 1.5 for healthy individuals. Notably, the highest average count was observed in the healthy group. This number progressively decreased as the severity of periodontal disease increased, as shown in Table 2 and Figure 4.

**Table 2:** Mean rugae numbers among healthy individuals, gingivitis patients, and patients suffering from chronic as well as aggressive periodontitis

Healthy ± SD	Gingivitis ± SD	Chronic periodontitis ± SD	Aggressive periodontitis ± SD	p-value
8.6 ± 1.5	8.03 ± 1.85	6.41 ± 1.43	6.65 ± 1.38	0.00138

**Figure 5:**
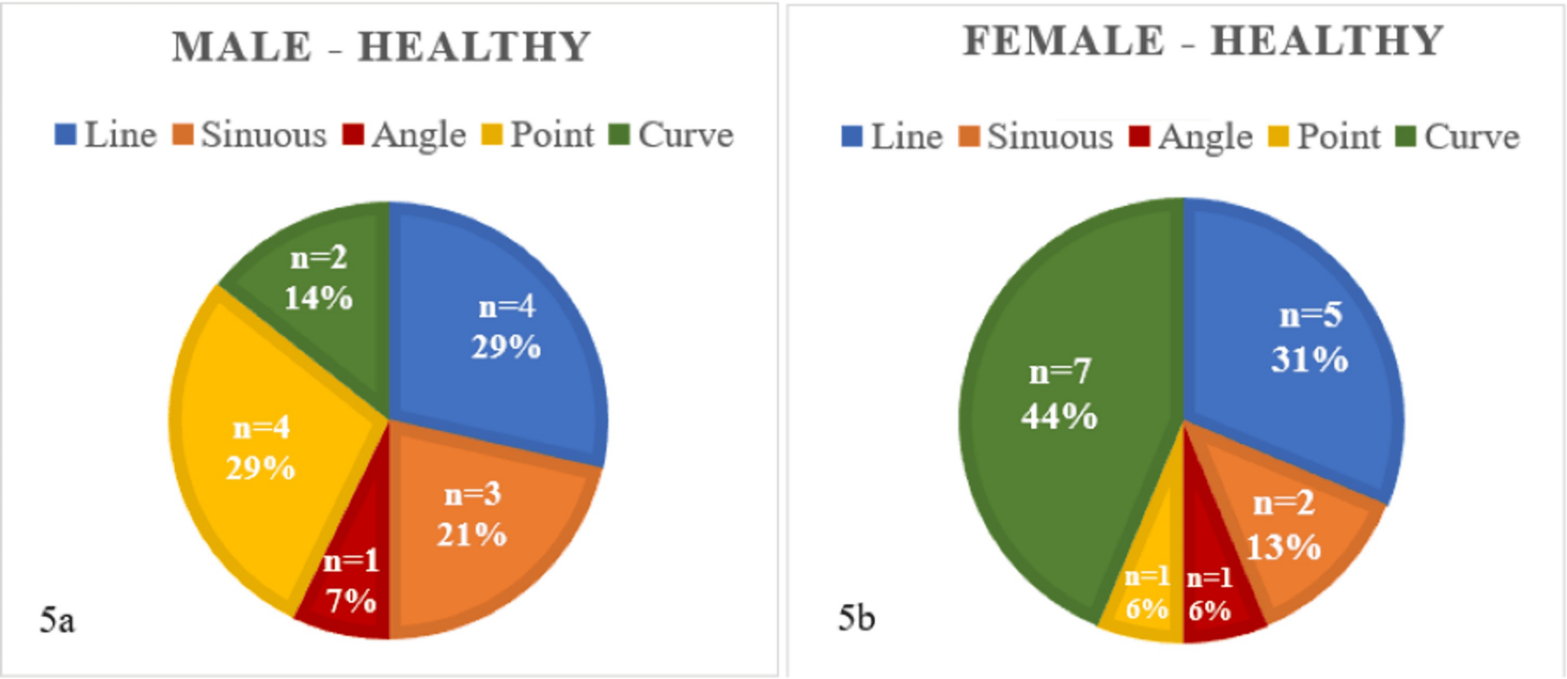
Depicts rugae patterns in male (5A) and female (5b) healthy individuals (n=30)

When evaluating correlations between gender and rugae pattern, it was observed that healthy females typically demonstrated a curved rugae pattern, followed by a line pattern. Conversely, healthy males displayed point and line patterns, as depicted in Figures 5a-5b. However, Table 3 indicates that there was an insignificant difference (p-value = 0.31) between rugae patterns in males and females in the given dataset of healthy individuals.

**Table 3:** Chi-square test of independence for healthy males and female subjects

Chi^2^ test for healthy individuals			
	Male	Female	Calculated p-value
Line	4	5	0.31
Sinuous	3	2	
Angle	1	1	
Point	4	1	
Curve	2	7	

**Figure 6:**
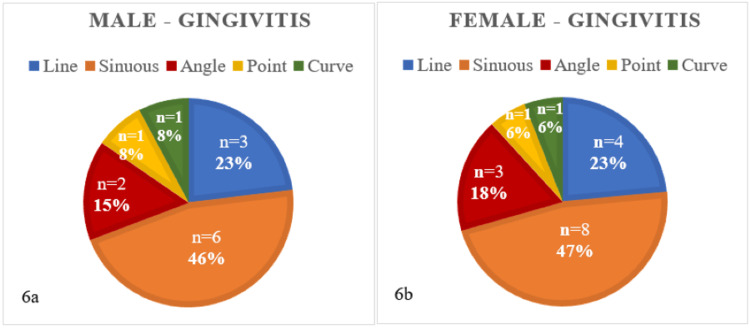
Rugae patterns in male (6a) and female (6b) patients with gingivitis (n = 30)

Among individuals with gingivitis, the sinuous pattern was prevalent in both males as well as females, as depicted in Figures 6a-6b. Table 4 indicates that there was an insignificant difference (p-value = 1) between rugae patterns in males and females in the given dataset of individuals with gingivitis.

**Table 4:** Chi-square test of independence for male and female subjects with gingivitis

Chi^2^ test for subjects with gingivitis			
	Male	Female	Calculated p-value
Line	3	4	1
Sinuous	6	8	
Angle	2	3	
Point	1	1	
Curve	1	1	

**Figure 7:**
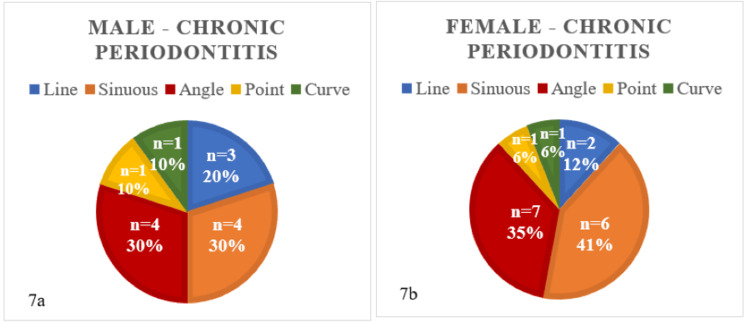
Rugae patterns in male (7a) and female (7b) patients with chronic periodontitis (n = 30)

Among males and females diagnosed with chronic periodontitis, there was a significant prevalence of sinuous and angular rugae patterns, as shown in Figures 7a-7b. Furthermore, Table 5 indicates that there was an insignificant difference (p-value = 0.88) between rugae patterns in males and females in the given dataset of individuals with chronic periodontitis.

**Table 5:** Chi-square test of independence for male and female subjects with chronic periodontitis

Chi^2^ test for chronic periodontitis			
	Male	Female	Calculated p-value
Line	3	2	0.88
Sinuous	4	6	
Angle	4	7	
Point	1	1	
Curve	1	1	

**Figure 8:**
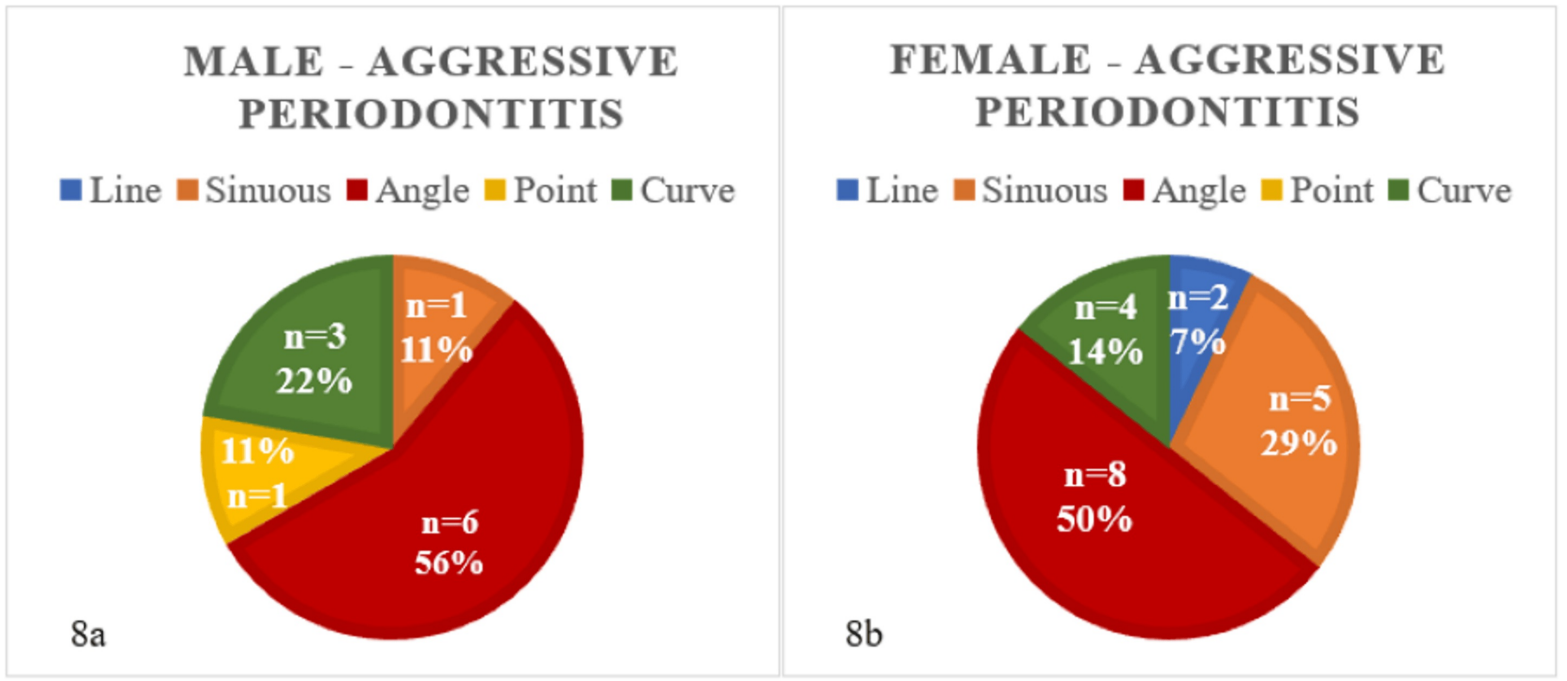
Rugae patterns in male (8a) and female (8b) patients with aggressive periodontitis (n = 30)

There was a greater occurrence of the angular pattern in both male and female patients diagnosed with aggressive periodontitis, while the line pattern was absent in males and the point pattern was absent in females, as depicted in Figures 8a-8b. Additionally, Table 6 indicates that there was an insignificant difference (p-value = 0.11) between rugae patterns in males and females in the given dataset of individuals with aggressive periodontitis.

**Table 6:** Chi-square test of independence for male and female subjects with aggressive periodontitis

Chi^2^ test for aggressive periodontitis			
	Male	Female	Calculated p-value
Line	0	2	0.11
Sinuous	1	5	
Angle	6	8	
Point	1	0	
Curve	3	4	

Our results challenge the notion of inherent gender-specific differences in rugae patterns and highlight their clinical relevance in relation to periodontal health. However, these findings highlight the hypothesis of a nuanced relationship between rugae patterns and specific periodontal conditions.

## Discussion

Palatal rugae consist of intricate ridges sustained by a core of hydrophilic glycosaminoglycan and dense connective tissue fibers. While identification methods like fingerprints, DNA analysis, and dental records have limitations in certain situations, palatal rugae have emerged as a widely utilized tool for population identification. In this study, calgurroscopy was selected as the analysis method for palatal rugae due to its simplicity and cost-effectiveness.

While several classification systems are available for analyzing rugae, Trobo’s shape-based classification system was specifically chosen for its simplicity in recording and lack of reliance on complex instrumentation [10]. Notably, the shape of rugae has demonstrated greater accuracy in classification compared to their length, making this system particularly suitable [11]. This study delved into both the shape and quantity of rugae, examining variations in their patterns between genders and differentiating between healthy individuals and those with periodontitis [12]. 

This study represents a pioneering effort to investigate the association between rugae patterns and gender, an area previously unexplored. We analyzed rugae patterns across four groups: healthy, gingivitis, chronic periodontitis, and aggressive periodontitis. To determine if rugae patterns were associated with gender, we compared between genders in each group by employing chi-square tests for independence. The chi-square statistics for the groups were as follows: healthy, p = 4.7768; gingivitis, p = 0.3110; chronic periodontitis, p = 0.882; significant differences in rugae patterns between males and females across all the groups. Conversely, our study identified a statistically significant (p = 0.00088), which is a significant (p < 0.05) association between rugae patterns and periodontal diagnosis.

A notable prevalence of linear and curved patterns is observed among healthy individuals, contrasting with a higher occurrence of sinuous rugae patterns in individuals with gingivitis. Chronic periodontitis patients exhibit a combination of sinuous and angular patterns, with sinuous patterns notably prominent on both sides of the rugae. Our research confirms that angular patterns predominate on both sides of the rugae in cases of aggressive periodontitis, closely followed by sinuous patterns.

In our study, we observed that the highest average count of rugae was found in the healthy group, with a progressive decline noted as the severity of periodontal disease increased. This is consistent with the research done by Sindgi et al. in 2018 [13], which reported a higher number of rugae in healthy normal individuals when compared to those suffering from periodontitis. When comparing genders, our study determined that males displayed a higher number of rugae than their female counterparts [14], which is in line with the findings of previous studies by Abrol et al. [15] and Sindgi et al. [13]. When evaluating rugae shapes between genders, our study found that healthy males typically exhibited point and line patterns, consistent with the findings of Jindal et al. [16] and Karnik et al. [17]. However, healthy females predominantly displayed curved rugae patterns, which contradict the results of the aforementioned studies.

Hermosilla et al. [18] utilized the Trobos classification and identified sinuous as the most prevalent rugae shape in periodontal disease, followed by other variations, including the curve, point, line, etc. This observation is further supported by Jindal et al., who determined the sinuous shape to be the most prevalent palatal rugae pattern in chronic periodontitis. Our findings corroborate with this trend, as sinuous patterns predominated in individuals affected by gingivitis and chronic periodontitis.

Our investigation revealed a prevalence of the angular pattern in both male and female patients diagnosed with aggressive periodontitis, consistent with findings from studies by Jindal et al. [16] and Abrol et al. [15]. Additionally, a notable finding from our study is the absence of line and point patterns in affected male and female patients. However, enhancing the sample size would strengthen the reliability of our findings.

The intragroup comparison indicates that there is no significant difference in the prevalence of rugae patterns between males and females. However, the intergroup comparison reveals a significant relationship between rugae patterns and periodontal diseases. This finding underscores the potential of rugae patterns as a diagnostic and monitoring tool for periodontal diseases.

Considering their shared genetic predisposition, each disease is associated with specific rugae patterns, which could potentially be used to predict the likelihood of a disease occurring in an individual with that particular pattern. This opens the door for a proactive approach to the treatment of periodontal diseases, shifting away from the current reactive methods. Hence, suggests that palatal rugae patterns could serve not just as a diagnostic tool but also as a potential risk predictor or marker for periodontal disease.

The study's limitations include its failure to investigate specific genetic markers or mechanisms that connect palatal rugae with periodontal disease. This omission restricts our understanding of how genetic variations might affect both rugae patterns and susceptibility to periodontal disease. Consequently, the study lacks a comprehensive explanation for the observed relationship between these variables. Moving forward, future research needs to incorporate genetic profiling to provide more profound insights into the genetic foundations of palatal rugae patterns and their association with periodontal disease.

## Conclusions

The discovery of correlations between palatal rugae patterns and distinct periodontal conditions presents captivating prospects for leveraging anatomical characteristics in diagnosing and treating periodontal diseases. Although rugae patterns are not gender-specific, they vary significantly among different periodontal conditions. Our findings underscore the significant clinical importance of rugae patterns, proposing their potential as a pivotal tool for the precise diagnosis and effective management of periodontal health. Utilizing palatal rugae as indicators for periodontal disease can facilitate early detection and predict its trajectory, thereby offering a promising avenue for effectively managing and potentially halting the progression of such conditions.

Continued advancements in this realm can elevate palatal rugoscopy to a sophisticated and precise diagnostic tool, surpassing traditional approaches and enabling comprehensive identification and understanding of diverse periodontal conditions.
